# Restoration of Impaired Metabolic Energy Balance (ATP Pool) and Tube Formation Potential of Endothelial Cells under “high glucose”, Diabetic Conditions by the Bioinorganic Polymer Polyphosphate

**DOI:** 10.3390/polym9110575

**Published:** 2017-11-04

**Authors:** Xiaohong Wang, Maximilian Ackermann, Meik Neufurth, Shunfeng Wang, Qiang Li, Qingling Feng, Heinz C. Schröder, Werner E. G. Müller

**Affiliations:** 1 ERC Advanced Investigator Grant Research Group at the Institute for Physiological Chemistry, University Medical Center of the Johannes Gutenberg University, Mainz, Duesbergweg 6, 55128 Mainz, Germany; mneufurt@uni-mainz.de (M.N.); Shunwang@uni-mainz.de (S.W.); hschroed@uni-mainz.de (H.C.S.); 2 Institute of Functional and Clinical Anatomy, University Medical Center of the Johannes Gutenberg University, Johann Joachim Becher Weg 13, D-55099 Mainz, Germany; maximilian.ackermann@uni-mainz.de; 3 Institute of Karst Geology, Chinese Academy of Geological Sciences, No. 50, Qixing Road, Guilin 541004, China; liqiang@karst.ac.cn; 4 Key Laboratory of Advanced Materials of Ministry of Education of China, School of Materials Science and Engineering, Tsinghua University, Beijing 100084, China; biomater@mail.tsinghua.edu.cn

**Keywords:** ATP pool, diabetes, tube formation, apoptosis, glucose, polyphosphate, endothelial cells, HUVEC

## Abstract

Micro-vascularization is a fast, energy-dependent process that is compromised by elevated glucose concentrations such as in diabetes mellitus disease. Here, we studied the effect of the physiological bioinorganic polymer, polyphosphate (polyP), on the reduced ATP content and impaired function of endothelial cells cultivated under “high glucose” (35 mM diabetes mellitus conditions) concentrations. This high-energy biopolymer has been shown to provide a source of metabolic energy, stored in its phosphoanhydride bonds. We show that exposure of human umbilical vein endothelial cells (HUVEC cells) to “high glucose” levels results in reduced cell viability, increased apoptotic cell death, and a decline in intracellular ATP level. As a consequence, the ability of HUVEC cells to form tube-like structures in the in vitro cell tube formation assay was almost completely abolished under “high glucose” conditions. Those cells were grown onto a physiological collagen scaffold (collagen/basement membrane extract). We demonstrate that these adverse effects of increased glucose levels can be reversed by administration of polyP to almost normal values. Using Na-polyP, complexed in a stoichiometric (molar) ratio to Ca^2+^ ions and in the physiological concentration range between 30 and 300 µM, an almost complete restoration of the reduced ATP pool of cells exposed to “high glucose” was found, as well as a normalization of the number of apoptotic cells and energy-dependent tube formation. It is concluded that the adverse effects on endothelial cells caused by the metabolic energy imbalance at elevated glucose concentrations can be counterbalanced by polyP, potentially opening new strategies for treatment of the micro-vascular complications in diabetic patients.

## 1. Introduction

Diabetes mellitus is a metabolic disorder, characterized by hyperglycemia that develops as a consequence of defects in insulin secretion [1]. One pathologic indicator of diabetes mellitus involves vasculature disturbances leading to both microvascular and macrovascular complications [2]. The global prevalence of diabetes has been estimated with 415 million (8.8%), with an increasing tendency; it is expected that, within the coming 25 years a rise to 642 million will take place [3]. The global economic burden of diabetes is enormous with about 11% of total health expenditure globally (US$548 billion) [4,5]. Major medications to treat diabetes involve insulin substitution or oral antidiabetic drugs, such as biguanides, sulfonylureas, meglitinide, thiazolidinedione, and dipeptidyl peptidase [6].

In the present study, it is proposed that normalization of the cellular ATP level positively affects the cells against “high-glucose” conditions, a main feature of diabetes mellitus. ATP has been well appreciated as the main intracellular energy store of the Gibbs free energy, which is sequentially released during enzymatic degradation of glucose to CO_2_ ([7,8]; reviewed in [9]). In addition, ATP acts as an extracellular signaling molecule by stimulating the purinergic transmissions [10,11]. The respective receptors have been classified into several groups, and P1 and P2 receptors are provided with individual selectivities for adenosine or distinct other purines or pyrimidines. There are the P2 receptors that are activated by purines/pyrimidines and by ATP; the subgroup of the P2X receptors represents ligand-gated ion channel receptors, while the P2Y receptors act as G protein-coupled receptors (reviewed in [12]). Usually the cells, such as osteoblasts or endothelial cells, are provided with multiple P2 receptor subtypes (P2X2,5,7) [13]; focusing on human umbilical vein endothelial cells (HUVEC cells), they express both P2X (predominantly subtype 7) and P2Y (subtype 11) receptors [14]. Recently, it has been proposed that polyphosphate (polyP) likewise interacts with the P2Y1 receptors resulting in an activation of phospholipase C and followed by the release of inositol 3 phosphate and an elevation of the Ca^2+^ level in the cytosol [15,16]. One potential result of this stimulatory effect on intracellular Ca^2+^ signaling is an increased production of ATP [16].

Experimental evidence has been provided disclosing polyP to provide high metabolic energy (Gibbs free energy), conserved within the phosphoanhydride linkages of the bioinorganic polymer, for the synthesis of ATP from its nucleotide (AMP or ADP) precursors [17,18]. polyP is a physiological inorganic molecule that has been identified both within cells, in the circulating blood [19], and in the synovial fluid [20]. The biopolymer is stored outside of the tissue in blood platelets. Within the cells, polyP is compartmented as particles in acidocalcisomes both in bacteria and metazoan cells (reviewed in [21]); it can be assumed that in these organelles polyP exists as amorphous microparticles. Based on this supposition, our group has prepared polyP microparticles, in the salt form of Ca^2+^-polyP, ranging in size from 80 to 250 nm [22,23]. The amorphous polyP particles retained their physiologically properties in the metazoan system [22]. Also fabricated as particles, polyP is prone to exohydrolytical cleavage via the alkaline phosphatase (ALP) [22,24]; other hydrolytic phosphatase(s) await to be discovered. During the enzymatic, hydrolytic, cleavage of the high-energy phosphoanhydride bond(s), the stored energy is liberated and transformed into other energy forms, surely again in metabolic energy under the formation of new covalent bonds [25]. This thermodynamic necessary consequence has been experimentally proven by the finding that mammalian cells exposed to polyP microparticles respond with an increased intracellular ATP pool [26]. Unpublished electron microscopical studies have shown that the polyP particles are taken up by cells [27], most likely via an endocytotic pathway [18].

In a previous study where HUVEC cells were used, we reported that the cell tube formation of endothelial cells is significantly upregulated after exposure to polyP [28]. Those HUVEC cells were grown on the physiological collagen scaffold. A commercially available collagen/basement membrane extract was used as a scaffold for the cells to grow onto. This effect has been attributed to an increased intracellular level of ATP as well as an inducing effect of polyP on gene expression, especially on the two major matrix metalloproteinases (MMPs), MMP-9 and MMP-2. Endothelial cells lining the blood vessels have crucial roles in a series of physiological/pathological events, like in vascular inflammatory responses. Often those adverse processes, caused by hyperglycemia, dyslipidemia, and/or hyperinsulinemia, result in impaired vasoregulation, oxidative stress, and inflammation—all processes that might cause vascular dysfunctions [29]. 

**Figure 1:**
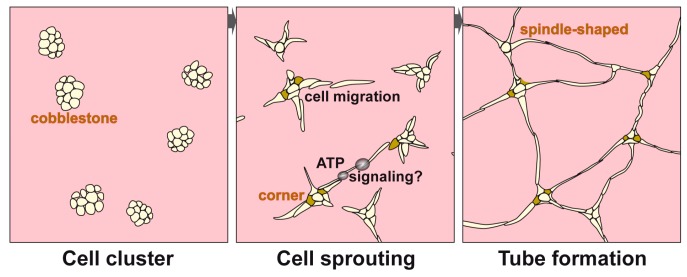
Schematic outline of the steps during tube formation by endothelial cells. First*:* Initially, cobblestones-like cells aggregate to cell clusters from which cells are sprouting out; Second*:* In those clusters cornerstone cells are formed which function as anchorage for the spindle-shaped cells; Third*:* Latter cells from adjacent clusters are guided chemotactically via an ATP gradient and join together to tubes.

It is established that the initial tube/microvessel formation, during the first 10 h, is based upon cell migration and association and occurs apparently in the absence of any cell division [27,28]. Cell migration happens on the fibrous collagen scaffold. As described here, the arrangement to tubes starts from cell clusters, composed of morphologically similar (cobblestone-like) cells. From those aggregates, cells sprout out to form spindle-shaped cells that connect neighboring cell clusters. These sprouting cells, forming web-like clusters of connecting tubes, are fixed with the cell clusters via trapezoid basic cells (cornerstones). It is surprising that (almost) no cells are found existing solitary on the basal membrane extract matrix, used for the tube formation. This observation implies that the cells, involved in the tube formation, migrate evidently with their motile intracellular (cytoskeleton) systems and their cell protrusions by the extension and retraction [30]. The non-directional cell migration will turn, during tube formation, to a directed movement theoretically by a series of chemo-attractants, like peptides/proteins (e.g., chemokines), small hydrophilic molecules (e.g., nucleotides), or bioactive lipids (e.g., endocannabinoids) [31]. From endothelial cells, it is known that they release ATP in dependence on their physiological and mechanical state [32], by mechanisms such as vesicular exocytosis, plasma membrane-associated ATP synthase, and ATP-binding cassette transporters. Pharmacological evidence suggests that the ATP stimulus is guided via purinoceptors (P2X4, P2X7, P2Y6, and P2Y12 receptors) and subsequently via PI3-kinase and Akt as well as via complement 5a to the dynamic elements of the cells [33]. It is interesting to note that ATP is rapidly present extracellularly (5–15 sec) after exposure to the suitable stimulus, a process that involves the H^+^-ATP synthase with its F1 catalytic domain [34]. Thus, we might propose that two spindle-shaped cells, originating from two opposite cell clusters, migrate by chemotaxis and join (together) via an ATP gradient, similar to that described for neutrophils, perhaps along with P2Y2 and A3 receptors [35]. These processes are summarized in the following scheme (Figure 1). 

In the present study, we addressed the question if polyP can restore the reduced ATP pool in cells that have been exposed to “high glucose” levels (>30 mM) in vitro. It is well established that under “high glucose” conditions in vivo, representing one major symptom of diabetes mellitus, adverse effects on endothelial cells lining blood vessels occur [36]. Two major consequences of “high glucose” exposure have been described in vitro; first, reduced intracellular ATP pool, and second, apoptotic cell death [37]. There are especially the reactive oxygen species that are generated in response to “high glucose” and are controlling these two processes [38]. One major crossroad, controlling the intracellular ATP pool, is located at the level of AMP-activated protein kinase (AMPK) [39]; this enzyme becomes activated when the intracellular AMP level increases and is inhibited at high AMP. Furthermore, activation of AMK inhibits the proteasomal degradation [40]. 

It is the aim of the present study to disclose the potential effect of polyP on microvascularization in vitro, using the tube formation assay, and the reduced intracellular ATP pool of “high glucose” exposed HUVEC cells. We describe that under “high glucose” conditions tube formation by HUVEC cells onto collagen is almost completely abolished. Even more, the cells under “high glucose” respond with an increased viability rate, especially after a >48 h incubation period. The adverse effects of increased glucose levels on cellular ATP content and ability of tube formation can be abolished by co-incubation with soluble polyP to almost normal levels. In a final series of experiments, it is shown that the level of intracellular ATP is positively correlated with cell survival. Our results reveal that polyP may have the potential to restore the “high glucose” compromised function of endothelial cells, growing onto a collagen scaffold in vitro and likely also in in vivo systems.

## 2. Materials and Methods 

### 2.1. Materials

Na-polyphosphate (Na-polyP) with an average chain length of 40 phosphate units was from Chemische Fabrik Budenheim (Budenheim, Germany). For the experiments described here, Na-polyP was complexed in a stoichiometric ratio (molar ratio) to Ca^2+^ of 2 (with respect to the phosphate monomer); abbreviated as “Na-polyP[Ca^2+^]” as described [41].

### 2.2. Endothelial Cell Tube Formation Assay

The commercial assay system was used (Thermo Fisher Scientific, Waltham, MA, USA) and the studies were performed as described in the instructions from the manufacturer and following a published procedure [42]. In this system HUVEC cells (from Lonza, Basel, Switzerland) were cultivated in EGM-Plus Growth Medium (with 5 mM glucose), containing supplements [43] at 37 °C with 5% CO_2_. For the experiments cells at passage <11 were used. The matrix, formed from collagen/basement membrane extract (Geltrex; Thermo Fisher Scientific; #A1413202) was layered into 12 wells plates (Corning/Costar-Sigma, Taufkirchen, Germany). The dishes were overlaid with 1 × 10^5^ cells/well in 400 μL of conditioned medium. Tube formation was checked during the first 10 h by reflection electron microscope (REM). 

### 2.3. Cultivation of HUVEC Cells

HUVEC cells (Lonza) were cultivated in endothelial cell medium, (EGM-2; Lonza) containing 2% fetal bovine serum (FBS) and vascular endothelial growth factor (VEGF) for rapid proliferation, as described elsewhere [44]. The cells were grown in this medium, containing 5.5 mM glucose, at “low glucose” conditions [45,46]. In a second series, the cells were cultivated under “high glucose” conditions, by an addition of 30 mM glucose (d-glucose; Sigma #G7021) reaching a final level of 35 mM [47]. 

To analyze tube formation, the cells were grown (seeding concentration 2 × 10^4^ cells/mL) in 6-well plates without basement membrane extract but onto collagen coated bottom plates (CELLCOAT-coated; Biocompare, South San Francisco, CA, USA). The cells were stained with Calcein-AM (#17783, Sigma, Taufkirchen, Germany) and inspected with a fluorescence microscope (Olympus, Hamburg, Germany) using the wavelengths 496 nm (excitation) and 520 nm (emission) [48]. 

For the cell viability assays, the cells were seeded in 96-well plates at a density of 1 × 10^4^ cells per mL and incubated for up to 72 h [49]. 

Co-incubation with polyP was performed with 0 µg/mL, 3 µg/mL, or 30 µg/mL of Na-polyP[Ca^2+^] (based on Na-polyP). 

### 2.4. MTT Viability Assay

The colorimetric methylthiazolyldiphenyl-tetrazolium bromide (MTT) assay was applied to assess the cell viability [50]. In brief, after incubation, the cells were incubated first with 1 μg/mL of MTT (2 h) and subsequently with 20% SDS in 50% dimethyl-formamide (Sigma; 24 h). The formazan grains were dissolved and the optical density was measured at 570 nm. The respective values were calculated as the percentage over the control (“low glucose” conditions; 100% survival).

### 2.5. TACS Assay

The TACS assay kit (R&D systems, Minneapolis, MN, USA; #4822-96-K) was used to quantitate the percentage of apoptotic cells after cultivation of HUVEC cells under different concentrations of polyP (Na-polyP[Ca^2+^]) and grown at “low glucose” and “high glucose” conditions. After the indicated incubation periods, the cells in the 96-well plates were fixed in formaldehyde, followed by incubation with proteinase K [51]. Then, the materials were labeled with terminal deoxynucleotidyl transferase for 60 min. After washing, the cells were incubated with streptavidin-HRP (1 h). Finally, the HRP enzyme reaction was performed with TACS-sapphire. After termination of the reaction with phosphoric acid, apoptotic cells in each plate were determined with a plate reader at 450 nm.

### 2.6. Scanning Electron Microscopy

Scanning electron microscopic (SEM) observations were performed with the SEM microscope ESEM XL-30 (Philips, Eindhoven, The Netherlands).

### 2.7. Determination of the Intracellular ATP Pool

For the determination of the ATP content in HUVEC cells, the enzymatic ATP luminescence kit (no. LL-100-1, Cayman Chemical, Ann Arbor, MI, USA) was applied, as described [26,52,53]. The cells were grown to a density of ≈75%. After ATP extraction [54,55], the amount of ATP was calculated, using a standard curve for given ATP concentrations. The values are given as pmol/10^3^ cells.

### 2.8. Statistical Analysis 

After verification that the respective values follow a standard normal Gaussian distribution and that the variances of the respective groups are equal, the results were statistically evaluated using the independent two-sample Student’s *t*-test [56].

## 3. Results

### 3.1. Endothelial Cell Tube Formation of HUVEC Cells onto Collagen/Basement Extract

**Figure 2:**
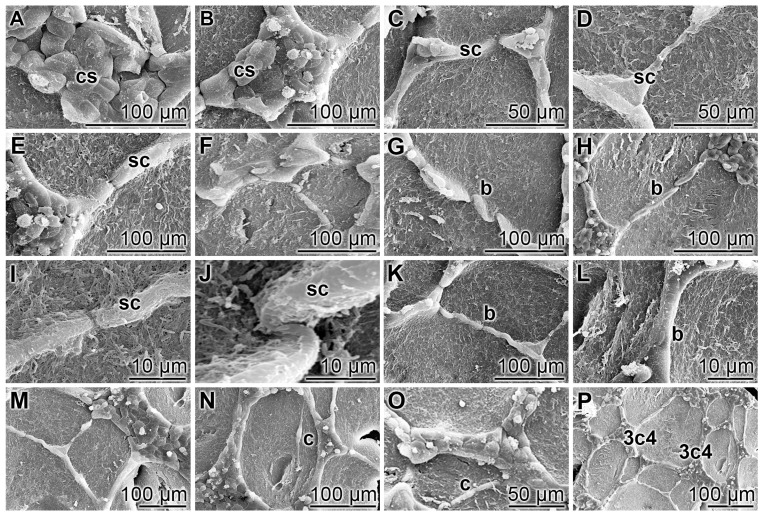
The HUVEC cell-based tube formation onto solubilized collagen/basement membrane extract was performed as described under “Materials and Methods”; SEM. Operationally, this process, which lasts about 10 h, can be dissected into three phases. (**A**–**D**) During Phase 1 (until 2 h), some cobblestone (cs)-appearing endothelial cells elongate to spindle-shaped sprouting endothelial cells (sc). They are based on polarized trapezoidal cells; (**E**–**L**) The subsequent phase (2 to 6 h) is characterized by joining of spindle-shaped endothelial cells under bridge (b) formation between two cell clusters; (**M**–**P**) In Phase 3 (until 10 h), a thickening of the cross web bridges develops by forming multilayer braces. During this process, a transversal apposition of scattered cells (c) occurs. Finally, interwoven tubes, built by three- to (rarely) four-rayed organization centers (3c4) form.

HUVEC cells undergo tube formation, if plated onto collagen/basement membrane extract, and growing in EGM-Plus Growth Media (Lonza) with supplements. This system contains 5 mM glucose, also termed “low glucose” conditions [57]. Under those conditions, the first tubes are completed already after 10 h (Figure 2). During this rapid process, no change of the cell number can be observed by eye-inspection. 

Phase 1: Within the first 2 h after seeding, some cells sprout out (Figure 2A–D) and elongate from initially approximately 25 to ≈50 µm. During this phase, the randomly arranged cells, with a cobblestone morphology, which form the initial cell aggregates (Figure 2A), become polarized and arrange in trapezoidal cell clusters. Those aggregates comprise three to four cornerstones (Figure 2B), which form the basis for the transversely arranged and spindle-shaped sprouting endothelial cells (Figure 2C); they protrude into the cell-free surrounding space. Frequently those sprouting cells associate together with outer sprouting cells, originating from another cell cluster. Initially those cells have no direct contact and secondarily communicate directly (Figure 2D). 

Phase 2: During the period of bridge formation, which occurs during the subsequent 4 to 6 h, two cell clusters interact via sprouting cells. Those bridges are, at the beginning, composed of two cells (Figure 2D,E). Later, additional cells become integrated into the bridges, building 3 to 4-cell crosspieces (Figure 2F,G). Of course, from the SEM observations, only hints about possible chemical signals, guiding those sprouting can be obtained. It is intriguing that in almost any case the sprouting cells from one cell cluster join the corresponding cells from a neighboring cluster (Figure 2H,I). Frequently, the corresponding sprouting cells slide across each other (Figure 2J), before they form a distal contact (Figure 2K). Occasionally, these sprouting cells wrap together (Figure 2L). 

Phase 3: The thickening, strengthening of the cross web in the way of a multilayer brace (Figure 2M–O) occurs by transversal apposition of apparently scattered cells (Figure 2N,O); 6 to 8 h after the start of the tube formation. Most final tubes that are formed during the first 10 h are multicellular in their bridges (Figure 2P). It might be stressed again that the pattern of interwoven tubes is built by three- to (rarely) four-rayed organization centers representing the initial cobblestone clusters (Figure 2P). 

### 3.2. Effect of Glucose Concentration on Tube Formation

**Figure 3:**
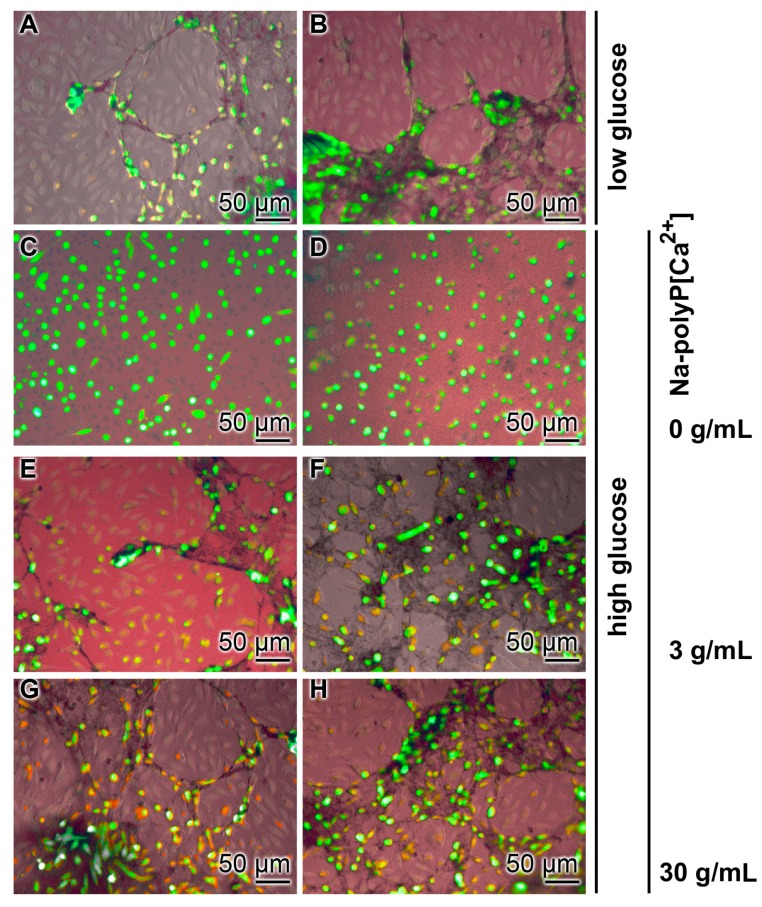
Inhibition of tube formation by HUVEC cells at “high glucose” conditions onto collagen coated bottom plates. The cells were incubated at (**A**,**B**) “low glucose” or (**C**–**H**) “high glucose” for 6 h (left panel) and 10 h (right panel). As indicated, the cultures grown under “high glucose” were supplemented with Na-polyP[Ca^2+^] at concentrations of 3 µg/mL or 30 µg/mL, respectively.

To elucidate the effect of glucose on tube formation, HUVEC cells were cultivated in EGM-2 medium in the absence of a collagen/basement membrane extract but onto collagen-coated bottom plates, as described under “Materials and Methods”. For contrasting, the cells were stained with Calcein-AM. At “low glucose” conditions, the first tubes formed after 6 h of incubation and were almost complete by 10 h (Figure 3A,B). In contrast, at “high glucose” the cells remained randomly scattered on the bottom of the well plates (Figure 3C,D). However, if those cells that were cultured at “high glucose” were supplemented with 3 µg/mL (Figure 3E,F) or 30 µg/mL of Na-polyP[Ca^2+^] (Figure 3G,H), a distinct association between the HUVEC cells and the tube-like assemblies was observed. This effect was observed in first outlines after 6 h, while at 12 h the tubes were almost closed. 

### 3.3. Reduced Cell Viability at “High Glucose”

**Figure 4:**
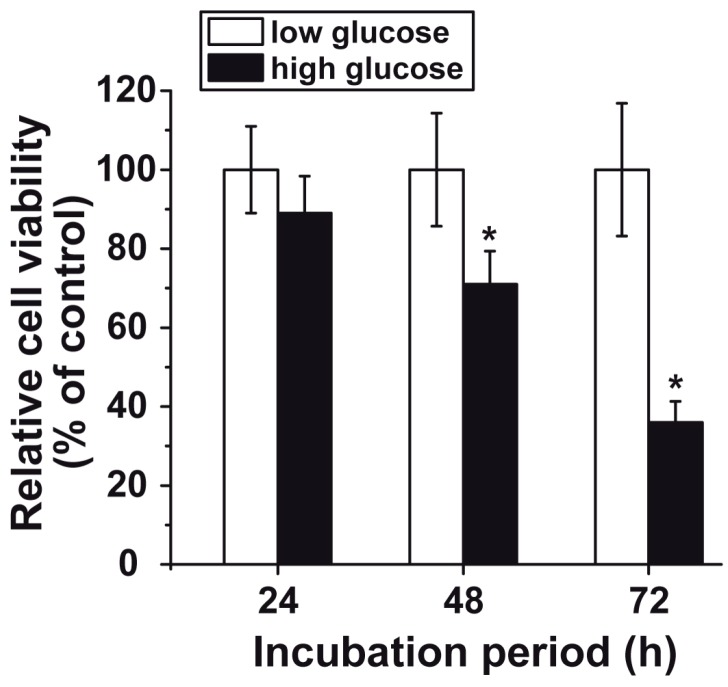
Viability of HUVEC cells measured during a 24 h-, 48 h-, and 72 h incubation period. The incubations were performed under “low glucose” (open bars) or “high glucose” (closed bars) conditions. The values are expressed as percent survival over control (incubation under “low glucose” conditions; set at 100%). The MTT reduction assay system was applied. The number of parallel experiments was 10. Data are means ± SD (* *p* < 0.05); the values are correlated to the corresponding numbers measured at “low glucose” conditions.

The HUVEC cells were incubated under the two different glucose concentrations in endothelial cell medium containing FBS and VEGF. After seeding, the cells (1 × 10^4^ cells per mL) started to proliferate and reached, under “low glucose” conditions, a concentration of 2.1 × 10^4^ (after 24 h), 2.7 × 10^4^ (48 h), and 3.4 × 10^4^ (72 h), respectively. If the cells were cultivated at “high glucose” concentrations, the relative cell viability decreased and reached a percentage of 89.3 ± 9.4% (after 24 h), 71.9 ± 8.4% (48 h) and 36.7 ± 5.3% (72 h), respectively, compared to the respective “low glucose” control. The latter two levels of inhibition are significant (Figure 4). 

### 3.4. Determination of Impaired Cell Viability as Apoptosis 

**Figure 5:**
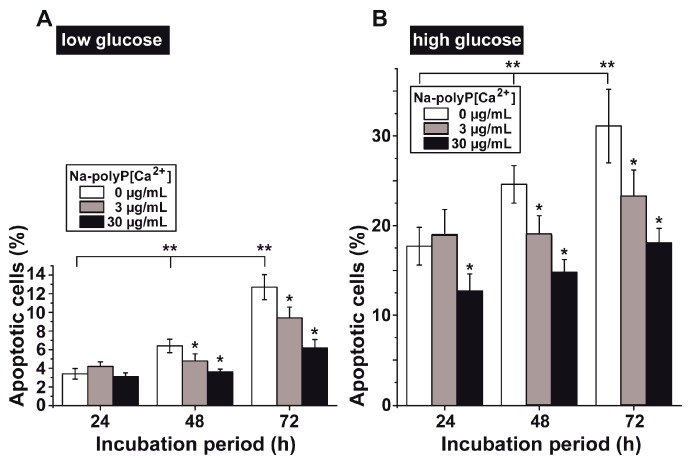
Reduction of the degree of apoptotic HUVEC cells after incubation without polyP (open bars), or with 3 µg/mL (grey) or 30 µg/mL of Na-polyP[Ca^2+^] (closed). The cells were incubated under (**A**) “low glucose” and (**B**) “high glucose” conditions. The percentage of apoptotic cells was determined by applying the DNA end labeling TACS assay kit. Ten parallel samples were performed per time point; data are means ± SD; * p < 0.05 are correlated to the values determined in assays without polyP. The significance between the values without polyP at a given time point and the controls is calculated as well and marked with ** p < 0.05.

In order to prove the possibility that this reduced viability is due to an increased rate of apoptosis, the results were evaluated using the terminal deoxyribonucleotide transferase apoptosis detection kit (TACS). This assay revealed that the percentage of apoptotic cells under “low glucose” was low and amounted to 3.4% (24 h), 6.4% (48 h), and 12.7% (72 h) (Figure 5A). If Na-polyP was co-administered, a significant reduction of the already low level of apoptotic cells was measured after 48 h and 72 h, respectively. We complexed Na-polyP with Ca^2+^ (Na-polyP[Ca^2+^]) in order to assure that polyP was not chelating out the calcium from the culture medium. 

Under “high glucose” conditions, the degree of apoptosis increased significantly to levels between about 20% and 35% (Figure 5B). Again, the percentage of apoptotic cells increased with the increasing incubation period, from 17.7 ± 2.1% (24 h) to 31.1 ± 4.1% (72 h). The addition of Na-polyP[Ca^2+^] caused a significant reduction in the number of apoptotic cells, even after the short 24 h incubation period, when 30 µg/mL of Na-polyP[Ca^2+^] was used. The effect of polyP was much more pronounced after a 48 h and 72 h incubation periods; a reduction was reached (after 72 h) by 25% at 3 µg/mL and by 42% at 30 µg/mL Na-polyP[Ca^2+^], respectively. 

### 3.5. Upregulation of the ATP Pool in Cells by polyP 

**Figure 6:**
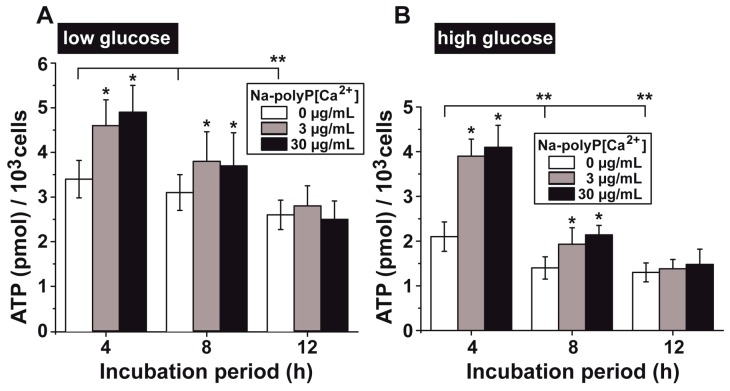
Effect of the glucose concentration in the medium on the ATP pool size in HUVEC cells. The cells were incubated under (**A**) “low glucose” or (**B**) “high glucose” conditions in the absence of polyP (open bars) or co-incubated with 3 µg/mL (grey) or 30 µg/mL of Na-polyP[Ca^2+^] (closed). Significant differences between the control values (minus Ca-polyP-MP) and the test sample (plus Ca-polyP-MP) are indicated (* p < 0.05). In addition, significant values between the controls and the test assays are indicated (** p < 0.05).

Under “low glucose” conditions, the intracellular ATP pool measured 3.43 ± 0.42 pmol/10^3^ cells after an initial incubation period of 4 h (Figure 6A). Over an extended period, the level dropped and reached the significantly different value of 2.68 ± 0.33 pmol/10^3^ cells after 12 h. The addition of Na-polyP[Ca^2+^] at concentrations of 3 µg/mL and 30 µg/mL increased the ATP level to levels that became significant after 4 h (increase by ≈40%) and 8 h (≈20%).

A similar ATP pool pattern was measured for the set of HUVEC cells, incubated at “high glucose” (Figure 6B). In comparison to the “low glucose” conditions, the ATP level was significantly lower, while the content after 4 h of incubation in “low glucose” amounted to 3.43 ± 0.42 pmol/10^3^ cells and dropped to 2.14 ± 0.33 pmol/10^3^ cells in cells grown at “high glucose”. If these “high glucose” cells were incubated with Na-polyP[Ca^2+^], a significant increase of the ATP pool was seen after both 4 h (increase by ≈90%) and 8 h (≈50%). 

## 4. Discussion

The morphogenetic process of micro-vascularization is a fast process that proceeds within a few hours or even minutes, since it requires no endothelial cell proliferation or differentiation [58]. It is a highly energy-consuming topological remodeling of the cells [59]. In patients with diabetes, wound healing and concomitant vascularization are impaired, resulting in a series of metabolic processes that are associated with the risks of developing cardiovascular disease, of rejection of transplanted organs, or of diabetic neuropathies [60,61]. Experimental studies have indicated that elevated glucose concentrations cause an increase in endothelial cell proliferation to a certain level, followed by an inhibition of cell function [62]. One major consequence is reduced ATP generation in the intracellular space but also on the cell surface [63,64]. The reduction of the intracellular ATP synthesis is caused by an increased accumulation of reactive oxygen species (ROS) followed by an impaired function of mitochondria in general and of the ATP synthesis in particular. The observed reduction of the ATP pool at the cell surface is associated with an increased activity of both the nucleoside triphosphate diphosphohydrolase and the ecto-adenylate kinase. The subsequent process of cell apoptosis can be prevented by an inhibition of the P2X7 purinergic receptor. 

Recently, we succeeded in demonstrating that the exposure of mammalian cells to polyP increases both the intracellular and extracellular ATP pool as well as the cellular ATP release into the cell environment [26,28]. The intracellular upregulation of the ATP level might be a result of an activation of the mitochondrial NAD kinase [16,65,66] and/or an increase in the number of mitochondria per cell [26]. In addition, experimental evidence has been presented suggesting that the enzymatic hydrolysis of the high-energy phosphoric anhydride bonds of polyP results in an increased extracellular ADP level, followed by an increased enzymatic synthesis of ATP via the adenylate kinase [28].

Now we show that if HUVEC cells are cultivated at “high glucose” levels onto collagen-coated surfaces, the viability of the cells decreases, the number of apoptotic cells increases, and the intracellular level of ATP drops. Those observations have already been published previously [36,37,38,39]. Importantly, in the present study we show that the number of apoptotic cells, not only under physiological “low glucose” but especially under “high glucose” conditions, is reduced to close to the normal level by Na-polyP concentrations between 3 µg/mL (30 µM) and 30 µg/mL (300 µM). These polyP concentrations meet the lower threshold of polyP, determined in mammalian tissue with 25 and 120 µM [67]. It should be stressed that, in our studies reported here, we used medium-size polyP, which has been shown not to activate apoptotic cascade (reviewed in [16]); in addition, the polyP sample applied was chelated with Ca^2+^ in order to prevent any membrane damage. 

As seen for the alteration of the viability of HUVEC cells in response to different glucose concentration, the intracellular ATP pool also undergoes a reduction at “high glucose” conditions in HUVEC cells. This pool returned to normal levels (“low glucose”) after exposure to Na-polyP. We explain this effect by the assumption that the reduced mitochondrial function under “high glucose” conditions, due to an increase in ROS [68,69], becomes normalized by polyP. 

Most likely as a consequence of the disturbed ATP balance in HUVEC cells exposed to “high glucose” concentration, an impaired ability of the cells to form capillary-like assemblies in the tube formation assay was observed. This effect was found to become abolished after co-incubation of the cells with polyP. Already at a low concentration of 3 µg/mL of Na-polyP[Ca^2+^], and even more pronounced at 30 µg/mL of the bioinorganic polymer salt, the HUVEC cells start to form tube-like structures after a short incubation period of 6 h.

Future studies must determine whether the polyP-mediated counterbalancing of the intracellular ATP level and the adverse response of the cells to “high glucose” is a direct effect of this biopolymer on the mitochondria, especially the membrane potential [70], or it is an indirect signaling effect via the cell membrane integrated P2Y1 receptor(s) [16]. Furthermore, it must be determined whether or not exposure of the cells to polyP modulates the intracellular load with reactive oxygen species, as indicated [71]. 

To summarize, in the present study we provide experimental evidence that endothelial cells, growing onto a collagen scaffold, respond to “high glucose” with the already described reduction of the cell viability as well as a drop of the intracellular ATP level. These two adverse metabolic reactions, as well as the impaired, energy-dependent tube formation, become normalized after exposure of the cells to physiological concentrations of polyP.
